# O.4.3-9 Perceived COVID-19 impact and its relationship with Physical Activity in youths in Luxembourg: Health Behaviour in School-aged Children 2022 survey

**DOI:** 10.1093/eurpub/ckad133.195

**Published:** 2023-09-11

**Authors:** Felipe Goedert Mendes, Joana Lopes Ferreira, Carolina Catunda

**Affiliations:** University of Luxembourg, Luxembourg; felipe.goedertmendes@uni.lu; University of Luxembourg, Luxembourg; felipe.goedertmendes@uni.lu; University of Luxembourg, Luxembourg; felipe.goedertmendes@uni.lu

## Abstract

**Purpose:**

The prevention measures undertaken due to the COVID-19 pandemic, such as lockdown and social distancing may have caused changes in the population’s lifestyle, including physical activity (PA). The current study aimed to investigate the PA level according to the perceived COVID-19 impact in youth in Luxembourg.

**Methods:**

This representative cross-sectional study was based on the Health Behaviour in School-aged Children (HBSC) Luxembourg 2022 survey. The population includes 8018 participants aged 11 to 18 years old. The PA was measured on how many days per week they practice 60 or more minutes of moderate-vigorous (MVPA) and vigorous PA (VPA). The perceived COVID-19 impact on PA was measured by the question “What impact did the Covid-19 measures have on your PA?”, with 5 answers categories ranging from very negative to very positive. A chi-square test was performed to observe the association between the perceived COVID-19 impact on PA and gender. In addition, a linear regression was performed to analyse the relation between MVPA and VPA and the perceived COVID-19 impact, adjusted by age, splited by gender.

**Results:**

An association was found between gender and the perceived COVID-19 impact on PA. 28.2% of boys perceived a very positive impacted of COVID-19 on PA, compared to 15.5% of girls. There was a relationship between MVPA, and the perceived COVID-19 impact on PA, in both genders. Participants who perceived a positive impact of the COVID-19 on PA practiced MVPA and VPA more frequently than the others, for both boys (β = 0.491, p < 0.001 and β = 0.491, p < 0.001, respectively) and girls (β = 0.543, p < 0.001 and β = 0.733, p < 0.001, respectively).

**Conclusions:**

Findings suggest a rather positive impact of the COVID-19 on PA, especially for boys. In addition, those who perceived a positive impact practiced significantly more days of MVPA and VPA, in both gender. This study highlights adolescent’s perception of COVID-19 impacts on their PA and draw attention to the positive impact perceived and its relation to an increased PA practice. Future studies should further investigate the reasons behind the perception of a positive impact, in order to mimic the contextual factors favouring the increased practice of PA.

